# High *HSPA8* expression predicts adverse outcomes of acute myeloid leukemia

**DOI:** 10.1186/s12885-021-08193-w

**Published:** 2021-04-29

**Authors:** Jun Li, Zheng Ge

**Affiliations:** 1 Department of Hematology, Zhongda Hospita, Medical School of Southeast University, Institute of Hematology Southeast University, Nanjing, 210009 China; 2 Hershey Medical Center, Pennsylvania State University Medical College, Hershey, PA17033 USA

**Keywords:** *HSPA8*, AML, Prognosis, Biomarker, Oncogene

## Abstract

**Background:**

Acute myeloid leukemia (AML) remains one of the most common hematological malignancies, posing a serious challenge to human health. HSPA8 is a chaperone protein that facilitates proper protein folding. It contributes to various activities of cell function and also is associated with various types of cancers. To date, the role of *HSPA8* in AML is still undetermined.

**Methods:**

In this study, public datasets available from the TCGA (Cancer Genome Atlas) and GEO (Gene Expression Omnibus) were mined to discover the association between the expression of *HSPA8* and clinical phenotypes of CN-AML. A series of bioinformatics analysis methods, including functional annotation and miRNA-mRNA regulation network analysis, were employed to investigate the role of *HSPA8* in CN-AML.

**Results:**

*HSPA8* was highly expressed in the AML patients compared to the healthy controls. The high *HSPA8* expression had lower overall survival (OS) rate than those with low *HSPA8* expression. High expression of *HSPA8* was also an independent prognostic factor for overall survival (OS) of CN-AML patients by multivariate analysis. The differential expressed genes (DEGs) associated with *HSPA8* high expression were identified, and they were enriched PI3k-Akt signaling, cAMP signaling, calcium signaling pathway. *HSPA8* high expression was also positively associated with micro-RNAs (hsa-mir-1269a, hsa-mir-508-3p, hsa-mir-203a), the micro-RNAs targeted genes (*VSTM4, RHOB, HOBX*7) and key known oncogenes (*KLF5, RAN*, and *IDH1)*, and negatively associated with tumor suppressors (*KLF12, PRKG1, TRPS1, NOTCH1, RORA).*

**Conclusions:**

Our research revealed *HSPA8 as* a novel potential prognostic factor to predict the survival of CN-AML patients. Our data also revealed the possible carcinogenic mechanism and the complicated microRNA-mRNA network associated with the *HSPA8* high expression in AML.

**Supplementary Information:**

The online version contains supplementary material available at 10.1186/s12885-021-08193-w.

## Introduction

AML (Acute myeloid leukemia) is a common type of myeloid neoplasms with highly heterogeneous clinical outcomes [1]. The majority of AML patients are with normal karyotype, some of these patients do well with chemotherapeutic consolidation, but others have a poor clinical outcome. Besides, the treatment of old or ineligible for receiving intensive chemotherapy remains a challenge [2, 3], as well as those relapsed and refractory AML patients [4–6]. Discovering new biomarkers for AML patients to stratify the prognosis system has been one of the hotspots in recent years. Cytogenetic normal AML (CN-AML) provided an ideal platform for biomarker recognition since no chromosome abnormalities exist.

Heat shock 70 kDa protein (HSP70) is reported to be involved in tumor cell proliferation and metastasis [7]. Heat shock 70 kDa protein 8 (HSPA8) is a member of HSP70 belonging to the heat shock protein family. It is a constitutively expressed molecular chaperone, which plays an integral role in cellular stress response [8]. *HSPA8* has been found overexpressed in various cancer cells, which was indispensable to the growth of cancer cells [7]. Furthermore, depletion of *HSPA8* could suppress cell growth, induce apoptosis, and cell cycle arrest in solid human tumors [9]. However, the role of *HSPA8* expression in AML is still poorly understood.

In this study, we identified a global gene expression profiling of *HSPA8* in AML patients. *HSPA8* was highly expressed in the AML cohort compared to the healthy cohort. The *HSPA8* high expression was significantly associated with the adverse prognosis. In addition, our study revealed that *HSPA8* expression might serve as an independent predictor for the OS of CN-AML, and *HSPA8* high expression was linked to cancer-related genomic alteration. The above findings correspond to the predictive capability of HSPA8 high expression in terms of 1-year and 3-year survival.

## Materials and methods

### Patients and sources

The first CN-AML cohort (*n* = 77) was derived from the TCGA dataset (*n* = 151) (detailed information in Additional file 5: Table S1), age ranged from 21 to 88 years of old, all of which were collected and diagnosed from Washington University between 2001 to 2010 [1]. Following the Declaration of Helsinki, all subjects provided written consent of the study and received traditional induction and consolidation regimen following the guideline of AML treatment. Samples were derived from the peripheral blood at the time of diagnosis. All information on the clinical, cytogenetic characteristics and survival information could be downloaded from the cancer genome atlas (TCGA). CN-AML patients with complete clinical data and RNA sequencing data were included in this study, AML-M3 patients were not included in this study.

Other AML cohort patients were collected from Gene Expression Omnibus (GEO)(GEO accession number: GSE12417 [10], GSE7186 [11], GSE9476 [12], GSE8970 [5], GSE5122 [6]). All patients also signed the written consent of the treatment and the study. Gene expression data and corresponding clinical characteristics could be downloaded from the GEO dataset.

Bone marrow samples of 32 healthy adult donors and 47 newly diagnosed adult AML patients (non-M3, 26 CN-AML patients) hospitalized from 2014 to 2019 from the hematology department, Zhongda Hospital of Southeast University (Nanjing, China) were collected for research. All patients signed with written informed consents in accordance with the Declaration of Helsinki before enrollment of the study.

### Reverse transcribed quantitative PCR (RT-qPCR)

The total RNA was extracted from bone marrow using Trizol agent (Invitrogen, USA) following the manufacture’s protocol. Then the mRNA was reverse transcribed into cDNA using the PrimeScript™ RT Master Mix (TaKaRa Dalian, China). The real-time PCR was performed on a StepOne Plus analysis system (Applied Biosystems 7300, Foster City, CA, USA), using TaKaRa SYBR Supermix (TaKaRa, Dalian, China). The amplification conditions of qPCR were as follows:95 °C for 30 s (pre-denaturation), 40 cycles of 95 °C for 5 s (denaturation) and 60 °C for 30 s (extension). The primers sequences were as follows:

- (*HSPA8*) Forward: 5′- CACTTGGGTGGAGAAGATTTTG-3′; Reverse: 5′- CTGATGTCCTTCTTATGCTTGC-3′. (*GAPDH*) Forward: 5′-GCAAATTCCATGGCACCGT-3′; Reverse: 5′- GACTCCACGACGTACTCAGC-3′.

The relative expression levels of the target genes were calculated by the 2^−ΔΔCt^ method.

### Bioinformatics analysis of transcriptome sequencing (RNA-seq) data

Differential expression analyses of the two groups (HSPA8^high^ and HSPA8^low^ group) from public datasets were conducted using the edgeR package. Benjamini and Hochberg’s approach were used to control the FDR (false discovery rate). Genes with an adjusted *P*-value < 0.05, FDR < 0.1 found by edgeR were considered statistically significant. The cluster profiler R package was used to assess DEGs’ statistical enrichment (differential expressed genes) in KEGG pathways. miRNA- mRNA regulation network was constructed on the Cytoscape platform based on the miRNA predicting targets (miRTarBase, miRDB, TargetScan datasets), common targets from the three datasets will be finally included.

### Statistical analysis

All the analyses were performed on the R 3.6.1 software platform. Data were presented as the mean ± SD. The overall survival (OS) was referred to as the time from the beginning of diagnosis to the death, induced by any causes. The correlation analysis of *HSPA8* and OS was estimated by the K-M (Kaplan-Meier) method. ANOVA method was used to compare several groups (> 2 groups). t-test and False Discovery Rate (FDR) was used to identify differences in sequencing profiles of coding and non-coding RNAs between *HSPA8*^high^ and *HSPA8*^low^ groups. Annotation files for microRNAs targets could be fully downloaded from the database of miRDB, miRTarBase, and TargetScan. The Cytoscape software was applied for the molecular networks. Data analysis was all conducted on the R 3.6.1 platform and Perl script.

## Results

### High expression of *HSPA8* is associated with adverse clinical outcome of AML

CN-AML patients derived from the TCGA dataset were divided into two groups according to the median expression level of *HSPA8* (CN-AML, cutoff value: 7.71(FPKM)). The baseline clinical and molecular characteristics of the two groups were compared in Table 1. There were no significant differences in terms of age, gender, bone marrow blast percentage, WBC counting, hemoglobin, platelet counting (*P* > 0.05) between the two groups. Genetic mutations (*KRAS, CEBPA, TET2, TP53, NRAS, IDH1*, *IDH2, RUNX1, NPM1*) were also presented with no significant difference, while the mutation frequency of *FLT3* increased significantly in the *HSPA8*^high^ group (*P* < 0.0001).

**Table 1:** Baseline patient characteristics according to the expression level of *HSPA8* in TCGA CN-AML patients

Group	Cohort		***P***
	***HSPA8***^**high**^	***HSPA8***^**low**^	
Total	38	39	
**Age**			
≥ 60y	22	20	0.649
< 60y	16	19	
**Sex**			
Male	18	24	0.256
Female	20	15	
**FAB classification**			
M0	0	6	0.036
M1	13	7	0.172
M2	13	7	0.172
M3	0	0	0.999
M4	4	15	0.01
M5	7	3	0.289
M6	0	1	0.999
M7	1	0	0.999
**Clinical characteristics** (mean(SD))			
BM blast (%)	42.84(33.17)	38.87(20.43)	0.792
WBC (×10^9)	36.62(36.52)	46.21(48.25)	0.334
Hemoglobin (g/L)	9.47(1.62)	9.92(1.35)	0.2
Platelet (×10^9)	80.46(66.98)	66.47(48.11)	0.303
**Mutation**			
*FLT3*	22	5	< 0.0001
*IDH1*	7	13	0.194
*IDH2*	4	8	0.227
*KRAS*	2	1	0.982
*CEBPA*	4	4	0.999
*TET2*	5	3	0.68
*TP53*	1	0	0.99
*NRAS*	2	2	0.999
*RUNX1*	2	9	0.056
*NPM1*	16	22	0.301

*FAB* French-American-British classification

**Fig. 1:**
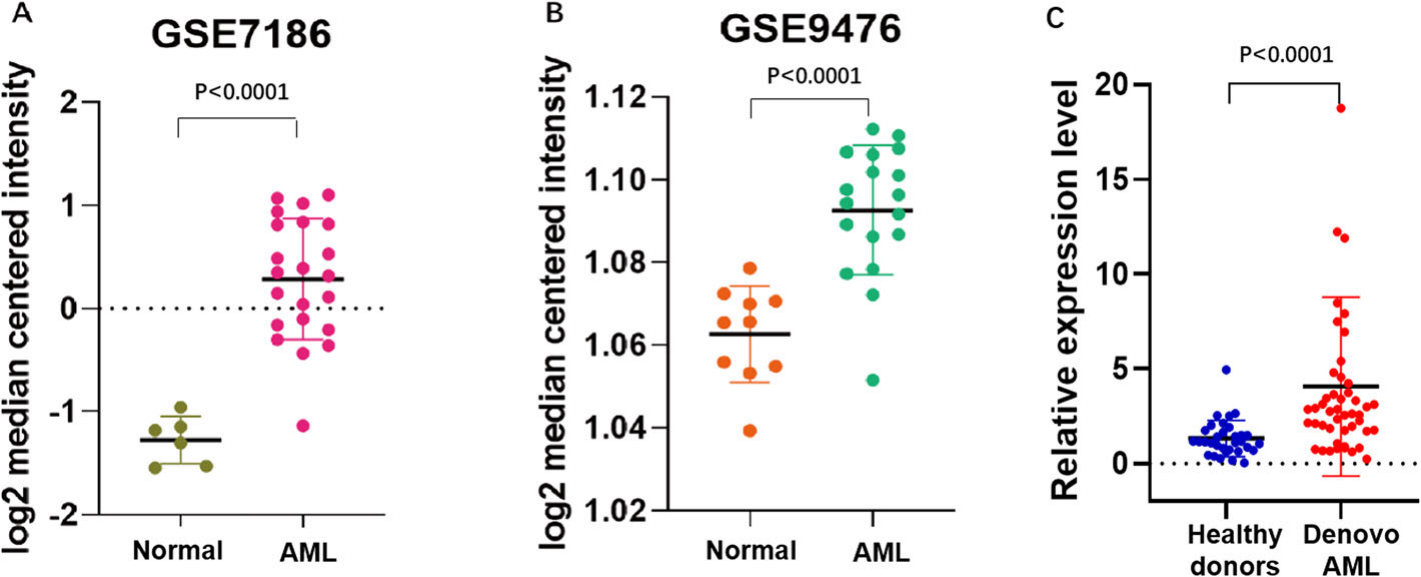
*HSPA8* high expression in AML compared to the healthy controls. Samples of GSE9476 were derived from peripheral blood (**a**), while samples of GSE7186 were derived from bone marrow (**b**). **c** The *HSPA8* mRNA expression level of bone marrow samples derived from patients hospitalized in Zhongda Hospital from 2014 to 2019(Nanjing, China) was statistically higher in de novo AML, than in the healthy cohort

Two datasets derived from the GEO were mined to compare the expression level of *HSPA8* between AML patients and healthy controls (GSE9476 and GSE7186). The result indicated that the HSPA8 mRNA level was significantly increased in the AML cohort compared to that of healthy peripheral blood (GSE9476) and bone marrow (GSE7186) controls (*P* < 0.0001) (Fig. 1a, b).

We further collected bone marrow samples of healthy donors and AML patients (non-M3) from Zhongda hospital (Nanjing, China) and examined the *HSPA8* expression to validate the above findings. The baseline data of 32 healthy donors and 47 AML patient were presented in Table 2. As expected, we observed a higher expression level of *HSPA8* in the de novo AML cohort, comparing to the healthy cohort (Fig. 1c) (*P* < 0.0001). Besides, we also observed a higher expression in the cytogenetic abnormal AML patients, though without significance (*P* = 0.1874) (Additional file 1).

**Table 2:** Univariate and multivariate COX analysis for survival analysis of TCGA CN-AML patients

Parameter	Univariate analysis			Multivariate analysis		
	HR	95%CI	***P***	HR	95%CI	***P***
**Age**	1.026	1.008–1.044	0.003	1.031	1.011–1.051	0.002
**Gender**	1.052	0.609–1.817	0.765			
**Bone marrow blasts**	0.996	0.983–1.009	0.507			
**WBC counting**	0.995	0.988–1.002	0.137	0.998	0.990–1.006	0.566
**Hemoglobin**	0.999	0.826–1.208	0.957			
**Platelet counting**	1.004	0.999–1.009	0.040	1.005	0.998–1.011	0.157
***FLT3*** **mutation**	2.6	1.461–4.631	0.001	1.881	0.920–3.848	0.084
***KRAS*** **mutant**	1.232	0.381–3.980	0.728			
***CEBPA*** **mutant**	0.624	0.248–1.572	0.317			
***TET2*** **mutant**	0.8	0.317–2.018	0.636			
***TP53*** **mutant**	1.938	0.264–14.248	0.516			
***NRAS*** **mutant**	0.24	0.033–1.742	0.158	0.282	0.036–2.234	0.231
***IDH1*** **mutant**	0.519	0.185–1.451	0.211			
***IDH2*** **mutant**	1.047	0.506–2.166	0.900			
***RUNX1*** **mutant**	2.22	1.089–4.524	0.028	2.233	0.969–5.145	0.059
***NPM1*** **mutant**	1.08	0.623–1.868	0.785			
***HSPA8***	2.042	1.068–3.907	0.031	2.408	1.080–5.368	0.032

**Fig. 2:**
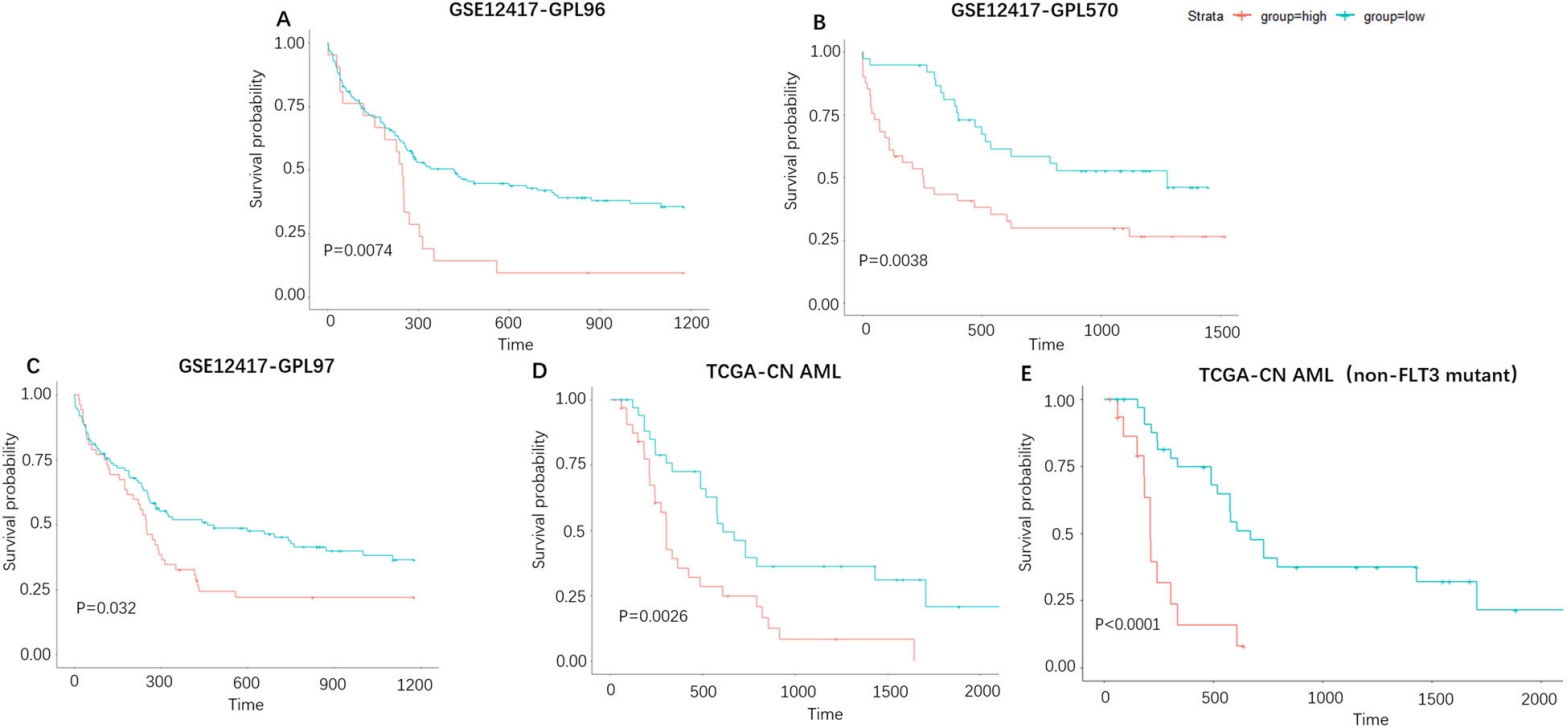
*HSPA8* acts as an adverse prognostic factor for CN-AML patients. **a** OS analysis of *HSPA8* in CN- AML group patients (GSE12417- GPL96 *HSPA8*^high^ group = 21, *HSPA8*^low^ group = 42); **b** OS analysis of *HSPA8* in CN-AML group patients (GSE12417- GPL570 *HSPA8*^high^ group = 41, *HSPA8*^low^ group = 38); **c** OS analysis of *HSPA8* in CN- AML group patients (GSE12417- GPL97 *HSPA8*^high^ group = 52, *HSPA8*^low^ group = 111); **d** OS analysis of *HSPA8* in CN- AML group patients (TCGA dataset, *HSPA8*^high^ group = 38, *HSPA8*^low^ group = 39); **e** OS analysis of *HSPA8* in CN- AML (non-FLT3 mutant) patients (TCGA dataset, *HSPA8*^high^ group = 16, *HSPA8*^low^ group = 34)

Kaplan-Meier survival analysis indicated that *HSPA8* high expression in CN-AML patients was significantly associated with the shorter OS (overall survival) (Fig. 2a-d, cutoff value for GSE12417-GPL570:11.73(FPKM), GPL96:12.46(FPKM), GPL97:12.97(FPKM)). Besides, an impaired survival benefit was observed among non-FLT3 mutant CN-AML patients (Fig. 2e). Furthermore, shorter OS was also observed in other AML groups such as cytogenetic abnormal, old AML, etc., not only the CN-AML group (Additional file 2A, B, C, D). The above findings indicated that the high *HSPA8* expression was significantly correlated with worse OS in AML.

### Univariable and multivariate Cox analysis of *HSPA8* in CN-AML patients

We next conducted univariate Cox analysis for 77 TCGA CN-AML patients, including 17 variables (*HSPA8* expression, age, gender, WBC counting, BM-blast, hemoglobin, platelet counting and genes mutation (*FLT3, KRAS, CEBPA, TET2, TP53, NRAS, IDH1*, *IDH2, RUNX1, NPM1,* mutation vs. wild type)). As is shown in Table 3, age, *FLT3* mutation, *RUNX1* mutation, platelet counting and *HSPA8* expression were associated with shorter OS (*HSPA8*, HR = 2.042, *P* = 0.031; age, HR = 1.026, *P* = 0.003; *FLT3* mutation, H*R* = 2.6, *P* = 0.001; *RUNX1* mutation, HR = 2.22, *P* = 0.028). Whereas other variables (*KRAS, CEBPA, TET2, TP53, NRAS, IDH1*, *IDH2, NPM1* (mutation vs. wild type), bone marrow blasts, WBC counting, hemoglobin, platelet counting) showed no statistical significance.

**Table 3:** Baseline patient characteristics of 32 healthy donors and 47 AML patients from Zhongda Hospital

	Cohort		***P***
	Healthy	AML	
**Total**	32	47	
**Age**			
**≥ 60y**	17	26	0.848
**< 60y**	15	21	
**Sex**			
**Male**	16	22	0.78
**Female**	16	25	
**FAB classification**			
**M0**	–	3	–
**M1**	–	5	–
**M2**	–	21	–
**M3**	–	0	–
**M4**	–	5	–
**M5**	–	9	–
**M6**	–	1	–
**M7**	–	3	–
**Karyotype**			
**Normal**	–	26	–
**Abnormal**	–	21	–

**Fig. 3:**
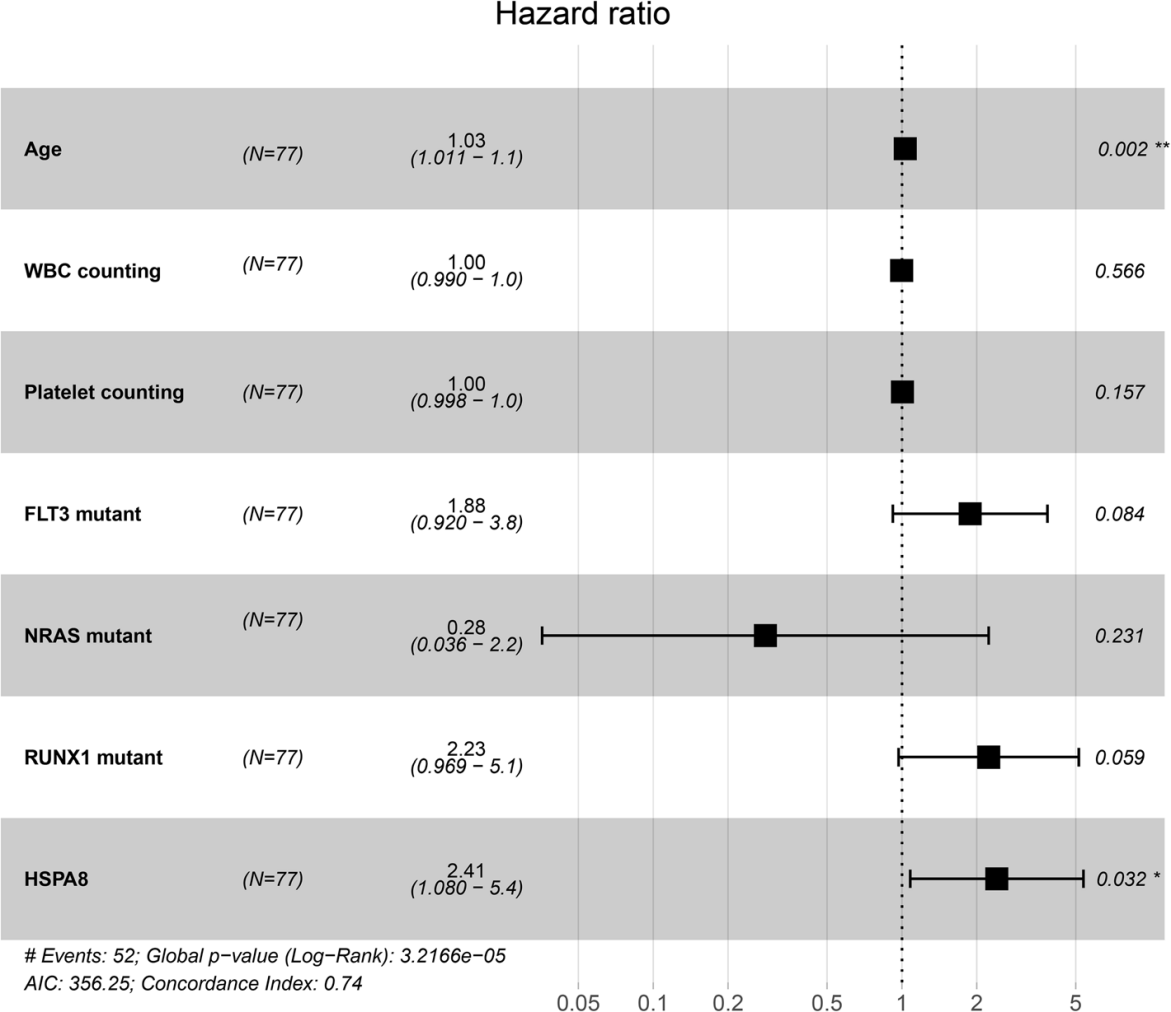
Multivariate Cox analysis for TCGA CN-AML patients. Age and expression level of *HSPA8* were presented to be independent factors for affecting survival (*P* < 0.05)

We further conducted the multivariate Cox analysis of OS (Table 3). Parameters with *P* value less than 0.2 during the univariate analysis were included in the Cox proportional hazards model. The result manifested that the age and *HSPA8* expression level were independent risk factors (Fig. 3) (*P* < 0.05). Besides, the high expression of *HSPA8* turned out to be the highest risk factor with an HR value of 2.408 (*P* = 0.032). Together, these results denoted that *HSPA8* high expression showed an independent risk factor affecting the survival of CN-AML patients.

### *HSPA8* high expression is a potential marker for shorter OS of CN-AML

**Fig. 4:**
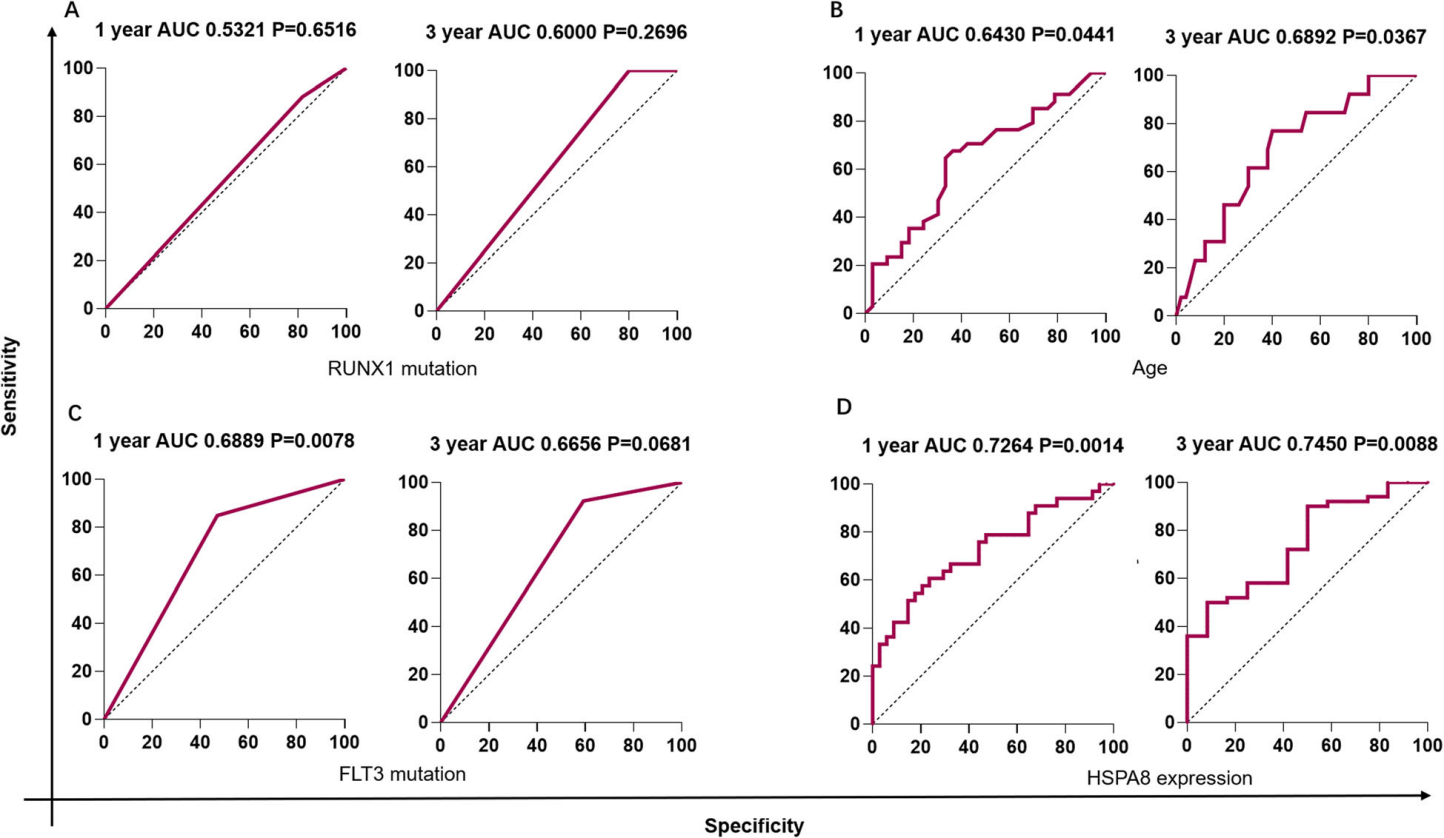
ROC curves of survival in TCGA CN-AML patients. The X-axis represents specificity, and the Y-axis represents sensitivity. ROC curves for gene mutation (*RUNX1* (**a**), *FLT3* (**c**), mutation vs. wild), age (**b**), *HSPA8* expression (**d**) in OS of TCGA CN-AML patients were performed. AUC-ROC of *HSPA8* expression reached 0.7264 (1-year survival, *P* = 0.0014), and 0.7450 (3-year survival, *P* = 0.00088), respectively

Based on the above results, we further hypothesized that the *HSPA8* expression could well predict the survival of CN-AML patients. Herein, we used AUC-ROC (Area under the receiver operating characteristic curve) of the survival model to investigate the potential prognostic value of *HSPA8* expression. The Youden index was calculated to dichotomize the cutoff point. The cutoff point in the 1-year survival data statistics is 7.812 (FPKM), while the 3-year survival cutoff point is 7.831; both were very similar to the median point of 7.71. The area under the ROC curve of *HSPA8* reached 0.7264 and 0.7450 in 1-year OS (*P* = 0.0014) and 3 years OS (*P* = 0.0088), respectively, indicating a better predictive performance, compared with other clinical variables (age, gene mutation (*FLT3*, *RUNX1*, mutation vs. wild type)) (Fig. 4). These results suggested that the high expression of *HSPA8* could serve as a valuable biomarker for predicting both the short- term and long- term survival of CN-AML patients. The result for *HSPA8* expression predicting other AML groups was presented in Additional file 3. Similar predictive capacity was observed in terms of 1-year and 3-year survival in old AML patients (age > 60 years old), but *HSPA8* had a lower predictive capacity for AML with karyotype abnormalities.

### Molecular characteristics and GO, KEGG pathways correlated with *HSPA8* expression

**Fig. 5:**
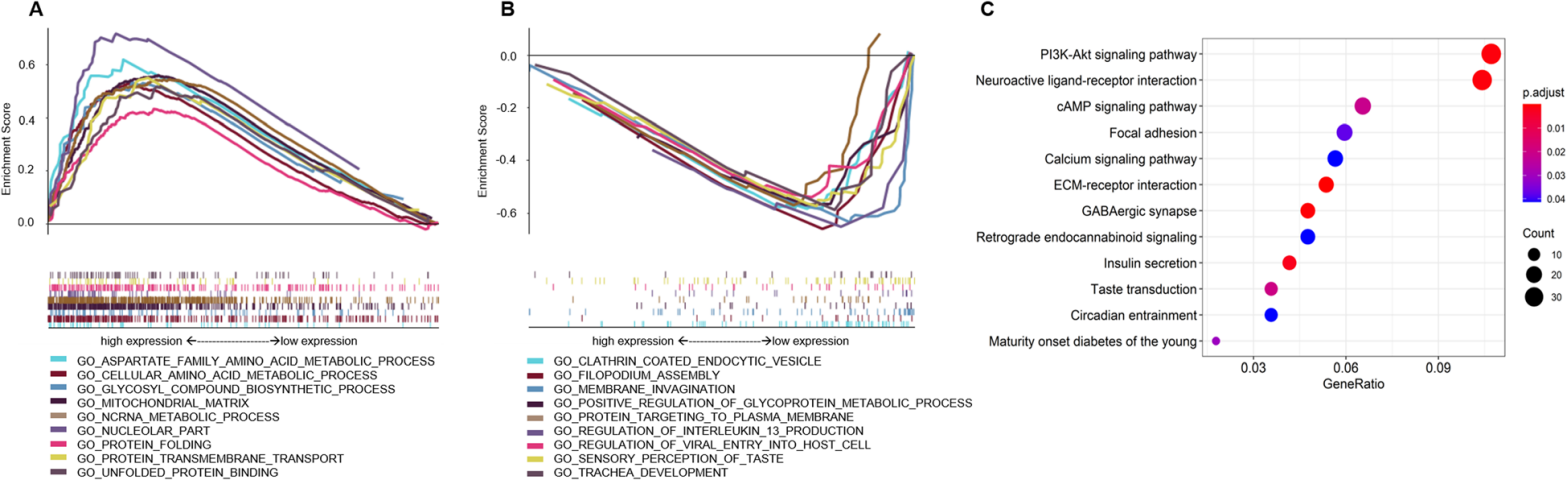
Function and pathway of *HSPA8* involved in the CN- AML. **a** Top 9 enriched GSEA-GO terms in the *HSPA8*^high^ group; **b** Top 9 enriched GSEA-GO terms in the *HSPA8*^low^ group; **c** Enriched KEGG terms of DEGs (differential expressed genes) (log_2_FC > 2.0, *p* < 0.01, FDR < 0.1) in the *HSPA8*^high^ group versus *HSPA8*^low^ group

To make a full overview of the role of *HSPA8* in the pathogenesis of AML, we identified the differential expressed genes (DEGs) using R software (edgeR package). Total 261 DEGs, including 131 upregulated genes and 130 downregulated genes, were identified (|log_2_FC| > 2, *P* < 0.01, FDR < 0.1) (Additional file 5). To delineate the role of *HSPA8* in the CN-AML, we carried out the gene ontology (GO) and Kyoto Encyclopedia of Genes and Genomes (KEGG) analysis. The GO analysis data revealed that the *HSPA8*-based gene-sets were significantly associated with multiple biological procedures, including protein folding, unfolded protein binding and metabolic process, etc. (Fig. 5a, b); the enriched gene-sets of GSEA result were presented in Additional files 7 and 8. The result of KEGG analysis indicated that *HSPA8*-based DEGs were significantly enriched in several critical signaling pathways, including the PI3k-Akt signaling, cAMP signaling, focal adhesion, calcium signaling pathway (Fig. 5c). The above results were validated by the GSEA analysis of a separate CN-AML dataset (GSE12417-GPL570, Additional file 4A, B).

### Genome-wide microRNA profiles linked to *HSPA8* expression

**Fig. 6:**
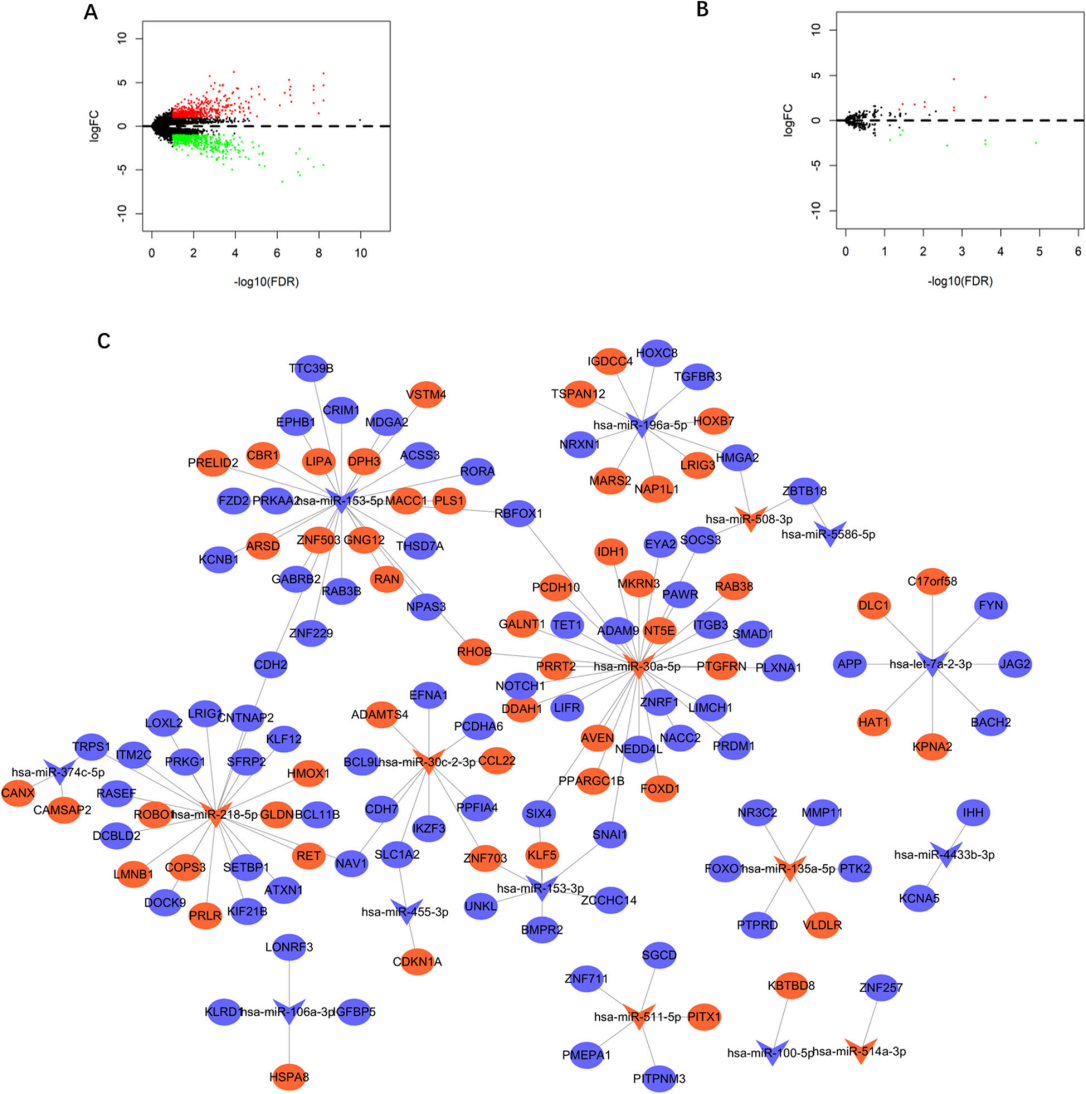
HSPA8 associated miRNA-mRNA network in CN-AML. **a** Volcano plot of differently expressed genes after comparing the *HSPA8*^high^ group with the *HSPA8*^low^ group among CN-AML patients. **b** Volcano plot of differently expressed miRNAs after comparing the *HSPA8*^high^ group with the *HSPA8*^low^ group among CN-AML patients. **c** miRNA- mRNA regulation and its correlation with *HSPA8*. Red nodes- upregulation in the *HSPA8*^high^ group; green nodes- downregulation

The association of *HSPA8* expression with the genome-wide expression profile was conducted based on the differential expressed micro- RNAs and DEGs (|log_2_FC| > 1, *P* < 0.01, FDR < 0.1) (Fig. 6a, b) to clarify the upstream regulation network. Finally, nine upregulated and seven downregulated micro-RNAs were included in this study. Three micro- RNAs (hsa-mir-1269a, hsa-mir-508-3p, hsa-mir-203a) were the most positively correlated with *HSPA8* expression.

Furthermore, we constructed the regulatory network of microRNA- mRNA network to clarify the biological functions associated with *HSPA8* expression. The network was analyzed by overlapping of the DEGs (including 53 upregulated genes and 83 downregulated genes) and the microRNA targeted mRNAs (|log_2_FC| > 1, P < 0.01, FDR < 0.1), based on the predicting algorithm (Fig. 6c).

Notably, *HSPA8*^high^ group was significantly associated with the upregulated level of many known oncogenes and the micro-RNAs’ predicted targets. Many oncogenes, including *VSTM4*, *RHOB, HOBX*7, were significantly upregulated in the *HSPA8*^high^ group. Other important oncogenes, including *KLF5, RAN*, and *IDH1*, were significantly upregulated in the *HSPA8*^high^ group. Meanwhile, tumor suppressors, including *KLF12, PRKG1, TRPS1, NOTCH1, RORA*, were downregulated in the *HSPA8*^high^ group.

## Discussion

The clinical outcome of cytogenetic normal AML (CN-AML) is significantly heterogeneous, accounted for nearly half of total AML patients [1, 13], which provided a good platform for the study of biomarker recognition since no chromosome abnormalities could be detected.

HSPA8 is a fascinating chaperone protein and plays an essential role in many biological processes [9]. Decreased expression of *HSPA8* is beneficial for suppressing the proliferation of cancer cells, inducing cell proliferation arrest, and acting as a modulator of viability and autophagy for cancer cells [9]. HSPA8 could enhance the stability of BCL2L11/BIM mRNA stability, which could regulate the total counts of hematopoietic cells with the help of BAG4, STUB1, HIP, HSP40 proteins [14]. Besides, *HSPA8* high expression has been identified in various cancer cells, including hepatocellular carcinoma and endometrial carcinoma, and is involved in cancer cell growth [15, 16] and regulating the autophagy in tumor cells [17]. Previous studies have reported that *HSPA8* can promote BCR/ABL-induced chronic myeloid leukemia cells’ survival [18].

In this study, we found that the expression level of *HSPA8* was higher in AML cohort than that in the healthy cohort. In addition, *HSPA8* high expression was associated with poor clinical outcomes in multiple AML datasets. Moreover, *HSPA8* high expression turned out to be the one of the highest risk factors related to a shorter OS by univariate and multivariate COX analysis and acted as an independent prognostic factor of the survival of patients with CN-AML. The AUC-ROC survival model analysis further revealed that *HSPA8* expression was presented as a valuable variable to predict the prognosis of CN-AML and old AML in terms of 1-year and 3-year survival. These findings highlighted the potential role of *HSPA8* in the pathogenesis of AML. Besides, we also observed a higher expression level among cytogenetic abnormal AML patients. This results indicated that the *HSPA8* high expression could be associated with adverse outcome of cytogenetic abnormal AML patients. However, more details need to be delineated in the future.

We further analyzed the predictive role of *HSPA8* expression in the survival of other AML subgroups. We found that it was not capable of predicting the survival for AML patients with karyotype abnormalities. However, a higher incidence of FLT3 mutation was found in *HSPA8*^high^ group patients. It is reported that a combination of HSPA8/HSC70 with HSP90 may form the HSP90/HSC70 protein complex, which remarkably enhances their ATPase activities [19]. Meanwhile, FLT3 protein and HSP90 could be included in a multiprotein complex [20]. These reports highlighted the potential relationship between FLT3 and HSPA8, the further details need to be further delineated in future studies.

We also investigated the correlation between the high expression of *HSPA8* and several oncogenic activities and signaling pathways, including PI3K-Akt and calcium signaling pathways. Specifically, constitutive PI3K activation was linked to reduced overall (OS) and disease-free survival (DFS) of 60% AML patients [21, 22]. Our data is consistent with the previous studies. For example, targeting the PI3k-Akt signaling pathway results in an anti-leukemic effect by activating oncogenes upstream (FLT3-ITD, KIT, NRAS, etc.) [23, 24]; and dysregulated Ca^2+^ homeostasis plays a crucial role in the pathogenesis of various cancers [25].

We further explored the possible underlying mechanisms for the high expression of *HSPA8* by analysis of the miRNA-mRNA regulatory network in CN-AML patients. The top deferentially expressed micro-RNAs were hsa-mir-1269a, hsa-mir-508-3p, and hsa-mir-203a. They were aberrantly expressed in various malignancies and played roles in tumorigenesis’s pathogenesis [26–28]. Interestingly, the hsa-mir-1269a and hsa-mir-203a were not present in the miRNA-mRNA network, underling the unknown regulatory mechanism or indirect regulation associated with the *HSPA8* expression, more details need to be clarified in the future. Besides, we did observe the reported/predicted targets of these miRNAs such as *RHOB*, *VSTM4*, *HOXB7* were increased in the patients. *RHOB*, the predicted target of hsa-mir-153-5p, was reported to stimulate relapse of the disease in *RUNX1-RUNX1T1* rearranged AML patients [29]. The ectopic expression of *VSTM4*, another target of hsa-mir-153-5p, could impair the anti-tumor immunity with robust T cell inhibitory activity [30]. Meanwhile, *HOXB7*, the target of mir-196a-5p, was overexpressed in various tumors and associated with a shorter OS [31].

We also observed that tumor suppressors, including *KLF12, PRKG1, TRPS1, NOTCH1,* and *RORA,* were downregulated in the *HSPA8*^high^ group. Notably, *TRPS1,* predicted targeted by hsa-mir-374c-5p and has-mir-218-5p, could inhibit *GATA* transcription [32], which could, in turn, prolong the OS of AML patients [33]. *NOTCH1,* the predicted target of hsa-mir-30a-5p, was downregulated in the *HSPA8*^high^ group; and overexpression of *NOTCH1* may result in the inhibition of AML cell growth in vivo. Another interesting finding is that the hsa-mir-153-5p has downregulated the expression of *RORA*, of which the increased expression is associated with the improved overall survival of AML [34].

These results together revealed the possible relationship of these miRNAs and transcription factors associated with *HSPA8* high expression and the complicated networks associated with *HSPA8* oncogenesis in AML.

In the clinical practice, the gene expression cannot be compared horizontally among multi- clinical centers, using standard quantitative RT-PCR or real time PCR method, based on the principle of relative quantification. One promising solution could be the application of digital-PCR, a third generation PCR for the quantification of gene expression, claimed to have unprecedented sensitivity, reproducibility and linearity [35, 36]. Herein, we believe it is necessary to launch a multi-center joint research to detect the expression of *HSPA8* in a large population, using digital-PCR method. Thus, it will be helpful to determine the diagnostic criteria of high *HSPA8* expression with multi-center clinical data which will serve as a foundation for further risk stratification.

In summary, we observed the *HSPA8* high expression in AML and have identified *HSPA8* high expression as a potential independent prognostic factor in CN-AML patients. *HSPA8* high expression is associated with cancer-related genomic alteration, including up- and downregulation of oncogenes and tumor suppressors, revealing the possible upstream signaling responsible for *HSPA8* high expression and downstream networks. Future studies will elucidate more details about in the function and oncogenesis of AML.

## Supplementary Information

**Additional file 1: Figure S1.** Comparison of *HSPA8* expression among different AML subgroups. (A) The *HSPA8* expression level of cytogenetic normal AML and cytogenetic abnormal AML. (B) The violin plot of *HSPA8* expression level among AML subgroups (5q-, 7q-, inv(16), t(15, 17), t(8, 21), t(9, 11), > 3 abnormalities, and cytogenetic normal type).

**Additional file 2: Figure S2.** *HSPA8* acts as an adverse prognostic factor for AML patients. (A) OS analysis of *HSPA8* in 34 relapsed/refractory AML patients (GSE8970 *HSPA8*^high^ group = 9, *HSPA8*^low^ group = 25) (*P* = 0.059). (B) OS analysis of *HSPA8* in 58 relapsed/refractory AML patients (GSE5122 *HSPA8*^high^ group = 7, *HSPA8*^low^ group = 51) (*P* < 0.0001). (C) OS analysis of *HSPA8* in cytogenetic abnormal AML patients (TCGA, *P* = 0.029). (D) OS analysis of *HSPA8* in 52 old AML patients (TCGA, age > 60s, non-M3, *P* = 0.0052).

**Additional file 3: Figure S3.** ROC curves of survival in AML patients. (A) 1-year and 3- year ROC analysis of *HSPA8* expression in old AML patients (age over 60s). (B) 1-year and 3- year ROC analysis of *HSPA8* expression in AML patients with karyotype abnormalities. The X-axis represents specificity, and the Y-axis represents sensitivity.

**Additional file 4: Figure S4.** Function and signaling pathway of *HSPA8* involved in the CN- AML (GSE12417-GPL570). (A) Top enriched GSEA-KEGG terms associated with the *HSPA8* expression. (B) Top enriched GSEA-GO terms associated with the *HSPA8* expression.

**Additional file 5: Table S1.** Clinical profiles of 151 AML patients derived from the TCGA dataset.

**Additional file 6: Table S2.** List of differentially expressed genes.

**Additional file 7: Table S3.** Enriched gene sets of GSEA analysis in *HSPA8*^high^ group.

**Additional file 8: Table S4.** Enriched gene sets of GSEA analysis in *HSPA8*^low^ group.

## Data Availability

The datasets generated and analyzed during the current study are available in the TCGA and GEO repository (https://portal.gdc.cancer.gov/;https://www.ncbi.nlm.nih.gov/geo/query/acc.cgi); Other datasets used and/or analyzed during the current study are available from the corresponding author on reasonable request.
