# Supplementary figures and images for: High HSPA8 expression predicts adverse outcomes of acute myeloid leukemia
Source: BMC Cancer. 2021 Apr 29;21:475. doi: 10.1186/s12885-021-08193-w (PMC8086305; doi:10.1186/s12885-021-08193-w)

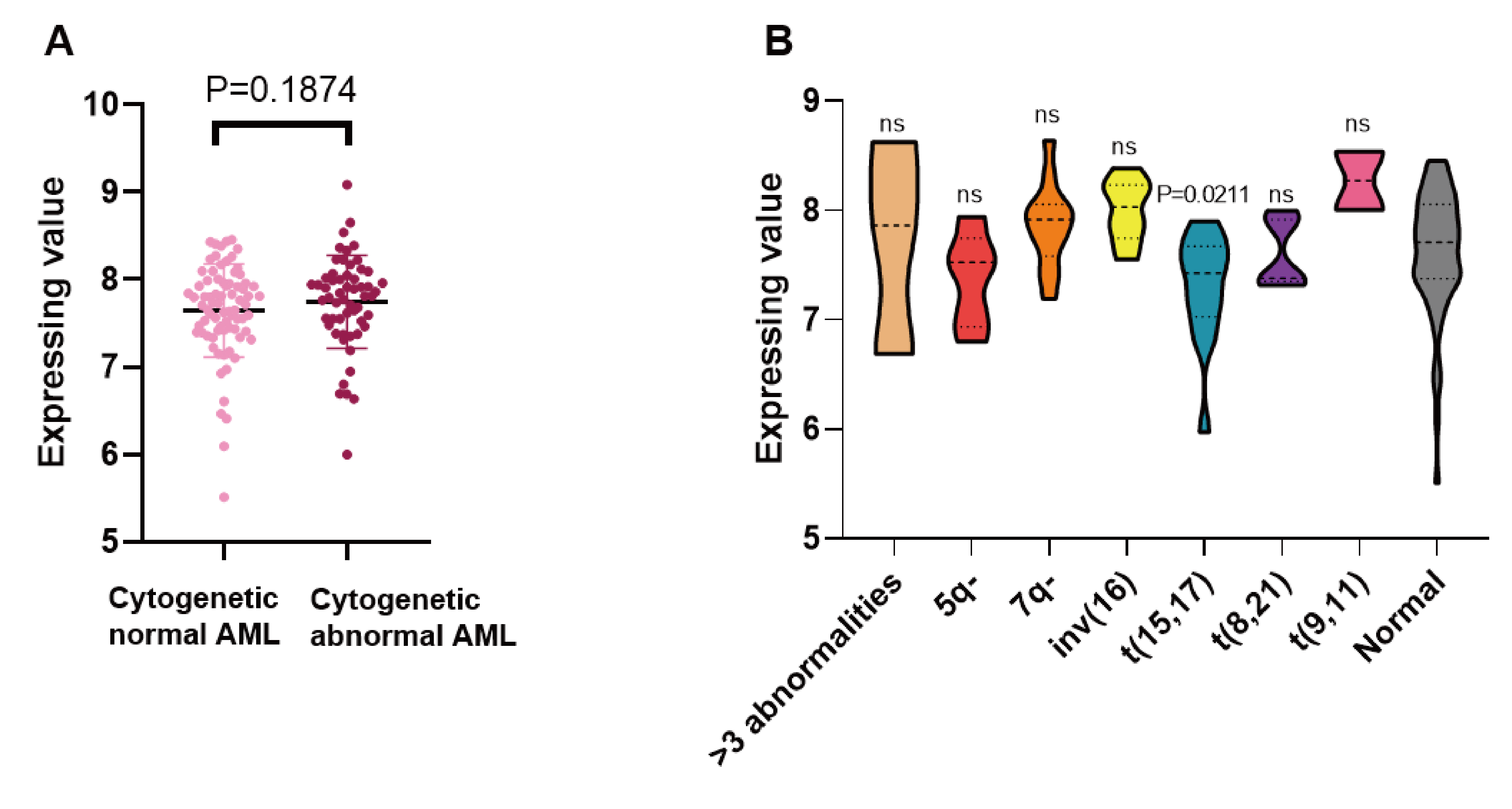

Supplement: Supplementary file 1 — Additional file 1: Figure S1. Comparison of HSPA8 expression among different AML subgroups. (A) The HSPA8 expression level of cytogenetic normal AML and cytogenetic abnormal AML. (B) The violin plot of HSPA8 expression level among AML subgroups (5q-, 7q-, inv(16), t(15, 17), t(8, 21), t(9, 11), > 3 abnormalities, and cytogenetic normal type). [file 12885_2021_8193_MOESM1_ESM.png]

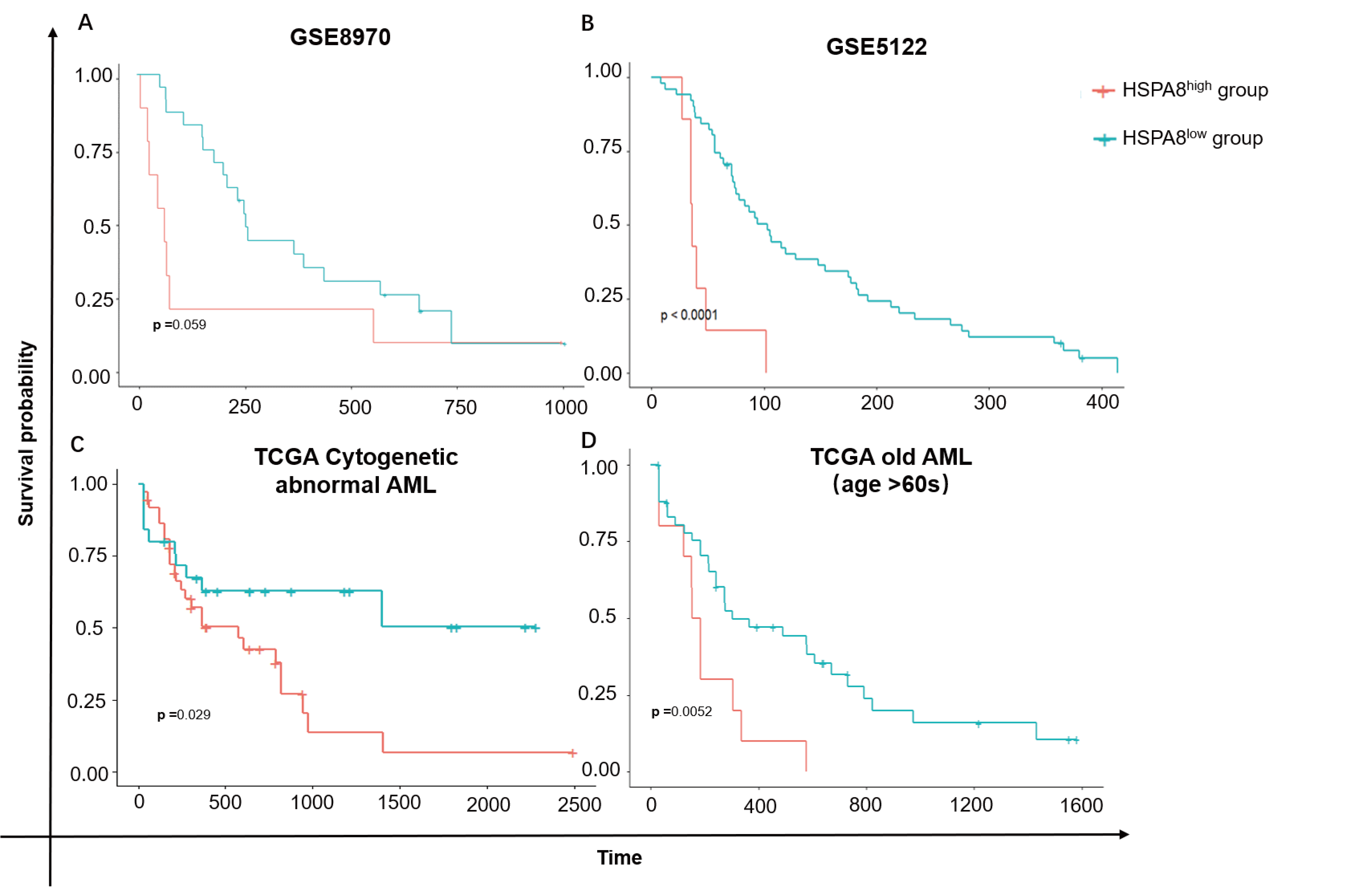

Supplement: Supplementary file 2 — Additional file 2: Figure S2. HSPA8 acts as an adverse prognostic factor for AML patients. (A) OS analysis of HSPA8 in 34 relapsed/refractory AML patients (GSE8970 HSPA8high group = 9, HSPA8low group = 25) (P = 0.059). (B) OS analysis of HSPA8 in 58 relapsed/refractory AML patients (GSE5122 HSPA8high group = 7, HSPA8low group = 51) (P < 0.0001). (C) OS analysis of HSPA8 in cytogenetic abnormal AML patients (TCGA, P = 0.029). (D) OS analysis of HSPA8 in 52 old AML patients (TCGA, age > 60s, non-M3, P = 0.0052). [file 12885_2021_8193_MOESM2_ESM.png]

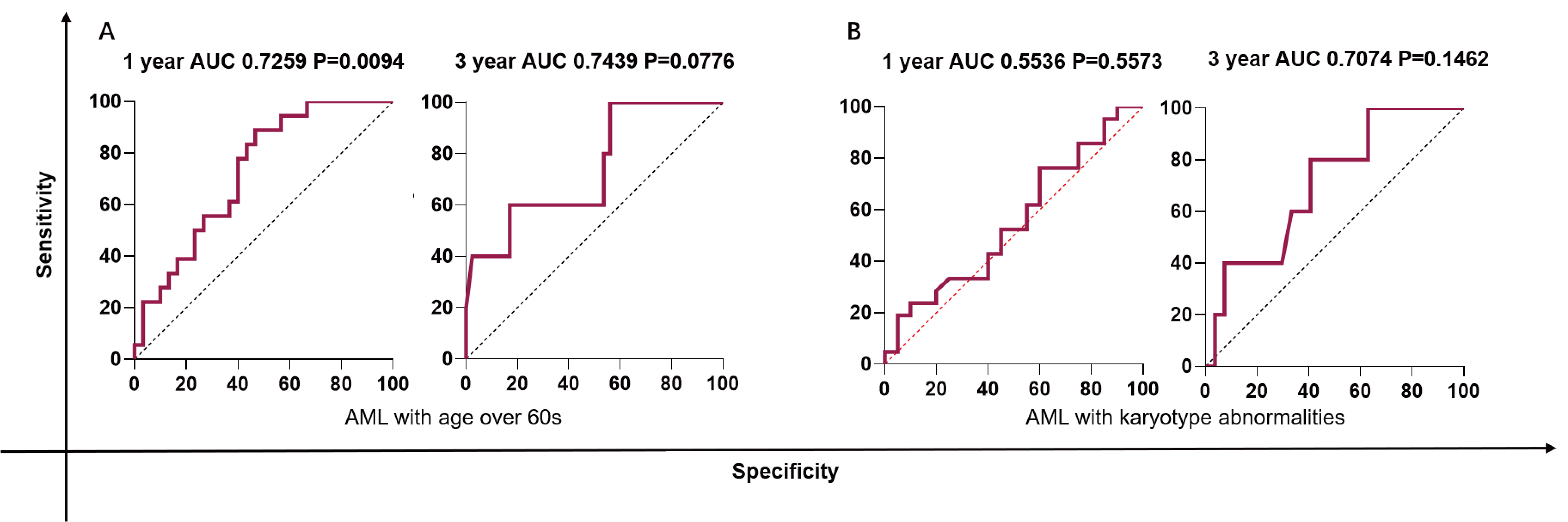

Supplement: Supplementary file 3 — Additional file 3: Figure S3. ROC curves of survival in AML patients. (A) 1-year and 3- year ROC analysis of HSPA8 expression in old AML patients (age over 60s). (B) 1-year and 3- year ROC analysis of HSPA8 expression in AML patients with karyotype abnormalities. The X-axis represents specificity, and the Y-axis represents sensitivity. [file 12885_2021_8193_MOESM3_ESM.png]

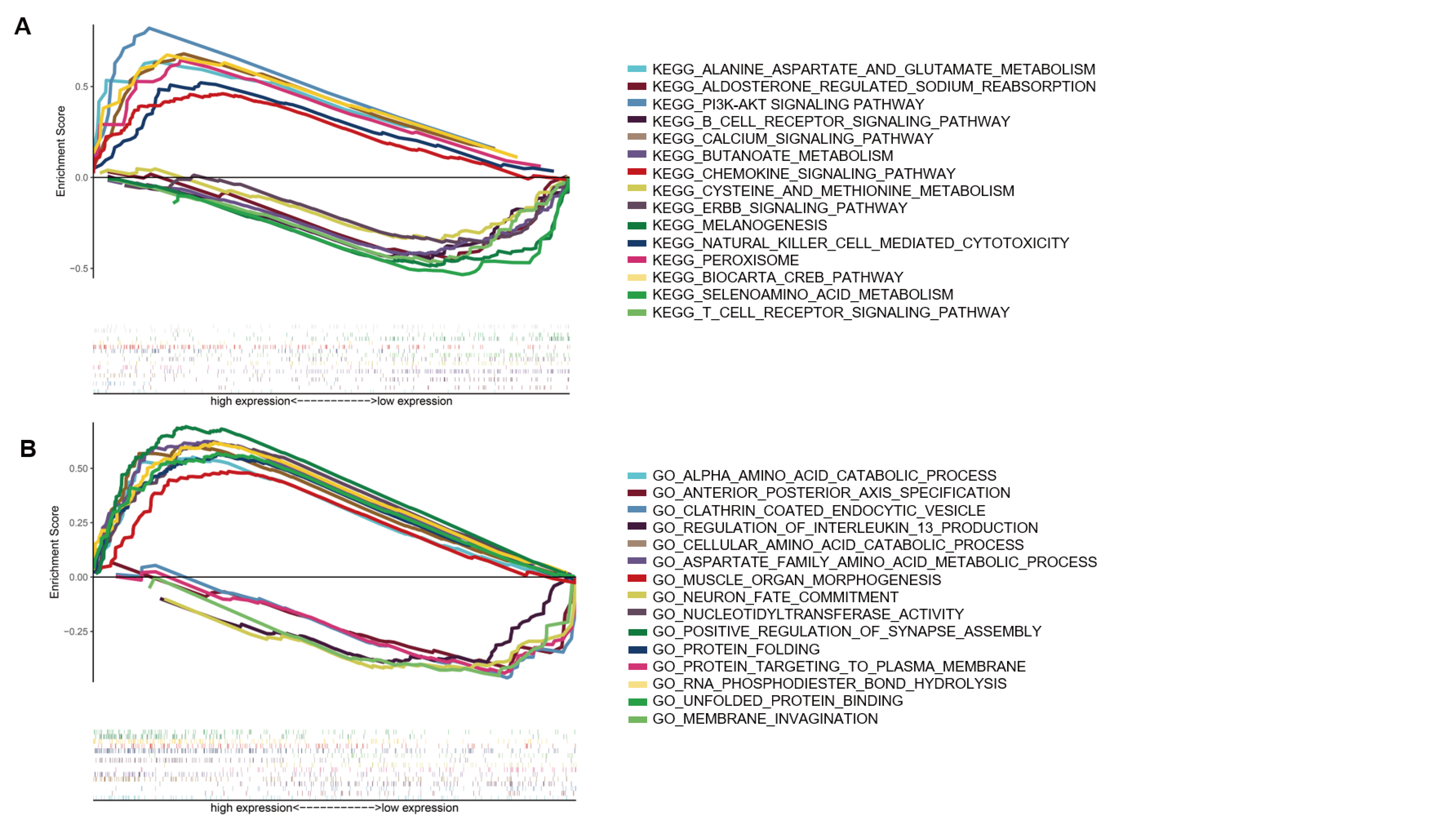

Supplement: Supplementary file 4 — Additional file 4: Figure S4. Function and signaling pathway of HSPA8 involved in the CN- AML (GSE12417-GPL570). (A) Top enriched GSEA-KEGG terms associated with the HSPA8 expression. (B) Top enriched GSEA-GO terms associated with the HSPA8 expression. [file 12885_2021_8193_MOESM4_ESM.png]
